# Endogenous and exogenous determinants of sex differences in blood pressure

**DOI:** 10.1038/s44325-026-00128-3

**Published:** 2026-05-25

**Authors:** Siyi Shangguan, Wasay Warsi, Juliane Louise Kwong, Yeran Lee, Susan Cheng

**Affiliations:** Department of Cardiology, Smidt Heart Institute, Cedars-Sinai Medical Center, Los Angeles, CA USA

**Keywords:** Physiology, Risk factors

## Abstract

Recent studies that have analyzed longitudinal data on blood pressure (BP) have led to a rapidly evolving understanding of sex differences in vascular aging phenotypes. We summarized the different BP trajectories in women and in men over the life course, and the accumulating evidence indicating that the effect of exogenous stressors, in conjunction with endogenous factors, impact BP trajectories to a larger extent in women than in men.

## Introduction

Cardiovascular diseases (CVD) often present differently in women and men, and such differences often become more apparent with aging^1^. Accentuated sex differences in CVD manifestations with aging are seen for various forms of myocardial dysfunction such as heart failure (HF) with preserved ejection fraction (HFpEF), for which cohorts enrolling elderly patients with mean ages 70–80 s demonstrate a female predominance^2–4^. Interestingly, even among healthy individuals, women over 50 years of age demonstrate steeper increases in cardiac filling pressures in response to saline volume loading when compared to women less than 50 years of age and when compared to men of any age group^5^. Studies have endeavored to identify the factors underlying the sex differences so that the treatment and prevention of CVD could be tailored to improve outcomes for both sexes. To date, our collective investigations have consistently led to the findings that such sex differences likely stem from intrinsic differences in anatomy and physiology, and in observed acute, chronic, and cumulative responses to cardiovascular stressors^6–9^. This understanding is still evolving, based on the growing evidence from experimental and clinical studies, including longitudinal cohort studies, many of which have tracked cardiovascular changes in both sexes with aging^10–12^. In particular, our understanding of sex differences in aging of the cardiovascular system is rapidly developing from recent numerous studies that have analyzed longitudinal data on blood pressure (BP)—a crude yet widely accessible measure of at least large artery systemic vascular integrity that has been assessed routinely across major studies in humans^12–14^. Herein, we will focus on BP and review both the endogenous factors (i.e., non-modifiable genetic and intrinsic hormonal) and exogenous factors (i.e., modifiable metabolic exposures and environmental stressors) that appear to influence BP trajectories and CVD outcomes differently in women and men over the life course.

## A brief overview of life course blood pressure trajectories

Our understanding of elevated BP as a vascular risk trait in the general population has improved tremendously over the last century. Whereas certain genetic factors can increase risk of hypertension particularly early in life, environmental factors are likely to confer cumulatively greater impacts on BP elevation over the life course. This conclusion is highlighted by the consistent observations from the International Cooperative Study on Salt, Other Factors, and Blood Pressure (INTERSALT study) that BP levels remain stable lifelong among indigenous among indigenous non-acculturated populations of Brazil, which have existed free from many of the vascular risk factors inherent to industrialized societies, such as excess salt intake^15,16^. Accordingly, data from the United Kingdom (UK) Biobank cohort have also demonstrated that features of a healthier lifestyle (i.e., balanced diet, limited alcohol consumption, low urinary sodium excretion, low body mass index [BMI], and increased physical activity) were strongly associated with lower midlife BP irrespective of underlying genetic risk^17^. Thus, it is now widely accepted that hypertension results from a complex interplay between genetic predisposition and environmental factors^18^. From re-analyses and cross-comparisons of age-specific BP data collected across various population-based cohorts over time, it is now clear that steadily rising BP is not a requisite feature of normal healthy aging as it was previously assumed^19,20^. It is also evident that, in the normal physiologic state, the healthy range for systolic BP is up to 10 mmHg lower in women than in men during youth and young adulthood^12,16,21^. Furthermore, we and others have reported on how, in the setting of usual exogenous exposures, age-related rise in BP appears to begin earlier and at a steeper rate of incline in women compared to in men^12–14,22–25^. The reasons for this sex difference are not yet defined. In addition to intrinsic differences in vascular anatomy, with women having smaller caliber vessels^26–28^, there are likely sex differences in a number of endogenous and exogenous factors that interact with aging.

## Endogenous determinants of sex differences in BP trajectories

We consider genetic and intrinsic hormonal differences as endogenous factors that not only determine sex differences in the observed normal range of BP in the healthy state, but that also contribute to sex differences in anticipated BP levels over the life course. At the outset, it is notable that the double dose of certain key genes on the X chromosome (e.g., angiotensin converting enzyme 2 [ACE2] and angiotensin receptor type 2 [ATR2] is thought to tilt renin-angiotensin-aldosterone system (RAAS) activity in favor of a vasodepressor phenotype in women while a single dose may favor a vasopressor phenotype in men^29^. In turn, the presence of certain loci (e.g., sex-determining region Y [SRY]) or variants on the Y chromosome (e.g., HindIII, which interacts with the aldosterone synthase gene cytochrome P450 family 11 subfamily B member 2 [CYP11B2]) may be a driver of elevated sympathetic activity and RAAS tone contributing to higher BP in men^30–32^. Sex differences in BP are influenced not only by sex chromosomal variants, as evidenced by genome-wide association studies having identified sex-specific BP traits in relation to autosomal variants^33^. In particular, certain autosomal gene variants appear to increase BP more so in women than in men (e.g., angiotensin receptor type 1 [ATR1] and beta-1 adrenergic receptor [ADRB1]) or increase BP more in pre-menopausal than in post-menopausal women (e.g., estrogen receptor 2 [ESR2])^34–37^. Notwithstanding these specific examples, the hundreds of gene variants associated with BP traits in sex-pooled genome-wide association studies have also been shown to demonstrate sex-specific relations with certain hypertension phenotypes^7,33,38^.

It is likely that many sex-specific genetic determinants of elevated BP are related to sex variation in hormonal activation over the life course. An abundance of studies have focused on puberty, menstruation cycles, and menopause, demonstrating the significant impact of female reproductive hormones on blood pressure regulation^39–42^. Physiologic BP levels appear similar by sex prior to puberty, then subsequently diverge in association with peri-pubertal rises in levels of estrogen, progesterone, growth hormone, Insulin-like Growth Factor-1, and insulin in girls^39,40,43–49^. Estradiol, in particular, is known to augment beta-adrenergic receptor^50^ activity and endothelial^48^ mediated vasodilation with BP-lowering effects. Estrogens may also inhibit vascular smooth muscle cell proliferation and neointima formation, thus helping to maintain arterial compliance^51^. Progesterone exerts a natriuretic and blood pressure-lowering effect by competing with mineralocorticoids^39,49^. Overall, estrogens and progesterone lower women’s BP in a time and context-dependent manner, and such an effect is gradually lost as women approach menopause. In contrast to estrogen’s protective role, androgens and primarily testosterone, appear generally counteractive to any efforts to maintain normotension. Mechanisms underlying these counteractive effects remain unclear and may involve renin-angiotensin-aldosterone system activation, renal impairment, or maladaptive gut microbiota pathways^52–54^. Interestingly and paradoxically, both low levels of androgens in men and high levels of androgens in women (as seen in polycystic ovary syndrome [PCOS]) are each associated with risks for obesity, elevated BP, and cardiovascular disease^55,56^.

**Fig. 1:**
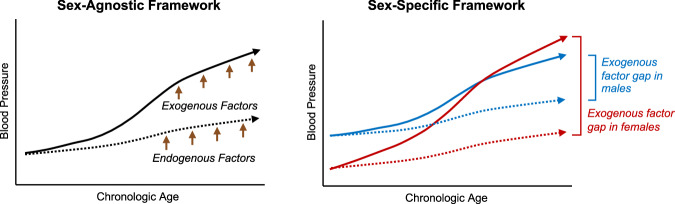
Blood pressure trajectories over the life course. The left panel demonstrates the blood pressure trends over the life course as affected by endogenous factors (dotted line) and exogenous factors (solid line) in the overall population; the right panel demonstrates the blood pressure trends over the life course as affected by endogenous factors (dotted line) and exogenous factors (solid line), among females (red) and males (blue).

Although the evidence in humans regarding the effects of sex-specific gene variants and hormone signaling pathways on BP trajectories is still in development, there are accumulating data linking genetic determinants of conditions such as hypertensive disorders of pregnancy (HDP) and PCOS with elevated lifetime risk of hypertension in women^57–60^. Similarly, there is growing evidence indicating that genetic traits predisposing one to premature menopause also increase the risk for later life hypertension consistently seen in estrogen and progesterone withdrawal^61^. Notwithstanding what are now commonly observed and widely presumed to be “acceptable” patterns of BP change in aging women and men, our understanding is still limited regarding why many and yet not all individuals demonstrate more accelerated BP elevation over the life course—a finding that remains more prominent in women than men (Fig. 1)^12,13,23^. For this reason, increasing attention is turned to the exogenous factors that add to and likely also interact with genetic and hormonal determinants of sex differences in age-related BP trajectories.

## Exogenous determinants of sex differences in BP trajectories

In contrast to endogenous factors, which are typically considered non-modifiable or minimally modifiable over the life course, we consider as exogenous factors the exposures and traits that are generally considered to be at least modestly to moderately modifiable. Exogenous factors include cardiometabolic risk traits such as obesity, diabetes, and dyslipidemia in addition to lifestyle and environmental factors such as dietary patterns, smoking, psychosocial stressors, toxins, and pollutants. Primordial prevention studies have long established that certain metabolic exposures can augment vascular risk that manifests as elevated BP or overt hypertension. We and others have found that BP response to metabolic stress exposures appears to be more exaggerated in women compared to in men. For instance, clinical and population studies have found that greater body mass index corresponds with a steeper BP rise in women than in men^62^. Similarly, type 2 diabetes is associated with a higher BP and tobacco product usage, including e-cigarettes, is associated with greater risk of hypertension in women than in men^63–65^. In our previously published study, 4 large community cohorts of more than 30,000 adults were followed longitudinally over four decades. We observed that a high cardiometabolic risk factor burden – including obesity, diabetes, dyslipidemia, and smoking - was associated with more accentuated BP elevation and greater overall BP load in women than in men^66^. A recent cohort study also suggested possible sex-differential BP responses to alcohol drinking cessation and initiation^11^. The extent to which endogenous factors interact with specific metabolic stressors to confer a greater impact on BP in women than in men is not yet well defined. However, it is evident that the sex differential BP response to stress exists and is not limited to metabolic exposures. Clinical and physiology studies have demonstrated that the known sensitivity of BP to sympathetic stress is greater in men than in women before the age of 40 years, but greater in postmenopausal women than in men of similar age ranges^67,68^. This age-dependent sex difference are supplemented by the mixed results in studies that have assessed BP responses to psychosocial or occupational stressors^69–73^. Notwithstanding the need for more prospective studies, existing data suggest that BP in women is likely more sensitive than in men to acute and chronic autonomic stress from a variety of sources—and particularly among postmenopausal women who appear most at risk for elevated BP and overt hypertension along with its associated clinical consequences^9^.

The mechanisms underlying greater BP sensitivity in females to certain exogenous stressors at various stages of life are not entirely clear, and likely attributable to multiple factors including but not limited to smaller caliber blood vessels subject to relatively greater dose exposures. For metabolic stressors, in particular, it is well established that the human anatomical and physiological traits intrinsically differ by sex even in the healthy state over the life course. For instance, women and men are known to differ in the quantity and distribution of subcutaneous and visceral fat as well as brown adipose tissue^74–76^. Therefore, intrinsic sex differences in multi-organ system substrate are likely an essential precursor to sex divergence in BP response to the variety of exogenous stressors that can be experienced over the life course.

Exogenous factors that can influence BP trajectories undoubtedly include medications and treatments that confer either intentional or unintentional sex-specific effects. Notwithstanding the influence of BP lowering medications, which are variably accounted for in sex-specific studies of BP elevation, exogenous hormone therapy prescribed to women is known to increase risk of hypertension if administered orally whereas alternate formulations (e.g., transdermal) may have neutral or even protective effects^77^. Data are also limited and equivocal regarding the BP effects of gender-affirming or cross-sex hormone therapy^78–80^, although recent studies suggest elevated BP risks among individuals receiving predominantly testosterone therapy and lower BP risks among those predominantly receiving estrogen and anti-androgen therapy^80–82^.

## Clinical implications and next steps

Given the aggregate evidence to date, we consider the effect of cumulative exogenous stressors, in conjunction with endogenous factors, as impacting BP trajectory to a larger extent in women than in men over the life course (Fig. 1). Should this conceptual framework continue to be supported by accumulating data in prospective studies, we are challenged with the question that whether certain exogenous stressors should be managed differently by sex and perhaps more aggressively in women. As of today, there are still insufficient prospective data to compel sex-specific clinical management of BP and associated risks^83^. While recent guidelines include consideration of female-specific conditions such as HDP in clinical care^84,85^, concerted efforts to investigate the effectiveness of more broadly applicable sex-based approaches may help address the remaining evidence gaps. As such endeavors continue, it is important to maintain a keen awareness of age-dependence of any presence or absence of sex-differential outcomes, including responses to therapies. For instance, a more aggressive approach to controlling elevated BP itself may demonstrate efficacy only early on, or at a younger age, beyond which the chronic BP elevation tends to correspond with arterial stiffening which is less reversible and may respond either non-differentially or unfavorably to more aggressive use of conventional BP-lowering therapies. Accordingly, particularly given continued global expansion of the aging population^86^, it is important to recognize the known potential harms associated with aggressive BP management in older patients depending on their increasingly diverse clinical risk-benefit profiles. For instance, whereas well-controlled BP is ideal for ameliorating cumulative risks in the setting of atrial fibrillation or heart failure, conventional approaches to achieving BP control may lead to unintended harm (e.g. syncope and falls) in the setting of certain age-related conditions, such as polypharmacy, frailty, and autonomic dysfunction with orthostasis^87^. By contrast, certain metabolically targeted agents such as glucagon-like peptide-1 receptor agonizts could offer favorable BP effects, particularly in women who are earlier rather than later in the course of experiencing an age-related rise in blood pressure. As ongoing investigations continue, ideally involving large prospective cohorts and broadly representative populations, our understanding will improve along with the abilities to more effectively tailor diagnostic, prognostic, and therapeutic approaches to vascular health for all.

## Data Availability

No datasets were generated or analysed during the current study.
